# Novel variants of the newly emerged *Anaplasma capra* from Korean water deer (*Hydropotes inermis argyropus*) in South Korea

**DOI:** 10.1186/s13071-019-3622-5

**Published:** 2019-07-25

**Authors:** Said Amer, Sungryong Kim, YoungMin Yun, Ki-Jeong Na

**Affiliations:** 1 College of Veterinary Medicine, Chungbuk National University, Cheongju, Chungbuk 28644 Republic of Korea; 2 Faculty of Science, Kafr El Sheikh University, Kafr El Sheikh, 33516 Egypt; 3 Chungbuk Wildlife Center, Chungbuk National University, Cheongju, Chungbuk 28116 Republic of Korea; 4 College of Veterinary Medicine, Jeju National University, Jeju, 63243 Republic of Korea

**Keywords:** *Anaplasma capra*, Korean water deer (*Hydropotes inermis argyropus*), South Korea

## Abstract

**Background:**

*Anaplasma* spp. are tick-borne Gram-negative obligate intracellular bacteria that infect humans and a wide range of animals. *Anaplasma capra* has emerged as a human pathogen; however, little is known about the occurrence and genetic identity of this agent in wildlife. The present study aimed to determine the infection rate and genetic profile of this pathogen in wild animals in the Republic of Korea.

**Methods:**

A total of 253 blood samples [198 from Korean water deer (*Hydropotes inermis argyropus*), 53 from raccoon dogs (*Nyctereutes procyonoides*) and one sample each from a leopard cat (*Prionailurus bengalensis*) and a roe deer (*Capreolus pygargus*)] were collected at Chungbuk Wildlife Center during the period 2015–2018. Genomic DNA was extracted from the samples and screened for presence of *Anaplasma* species by PCR/sequence analysis of 429 bp of the *16S* rRNA gene marker. *Anaplasma capra*-positive isolates were genetically profiled by amplification of a longer fragment of *16S* rRNA (*rrs*) as well as partial sequences of citrate synthase (*gltA*), heat-shock protein (*groEL*), major surface protein 2 (*msp*2) and major surface protein 4 (*msp*4). Generated sequences of each gene marker were aligned with homologous sequences in the database and phylogenetically analyzed.

**Results:**

*Anaplasma capra* was detected in blood samples derived from Korean water deer, whereas samples from other animal species were negative. The overall infection rate in tested samples was 13.8% (35/253) and in the water deer the rate was 17.8% (35/198), distributed along the study period from 2015 to 2018. Genetic profiling and a phylogenetic analysis based on analyzed gene markers revealed the occurrence of two distinct strains, clustered in a single clade with counterpart sequences of *A. capra* in the database.

**Conclusions:**

*Anaplasma capra* infection were detected in Korean water deer in the Republic of Korea, providing insight into the role of wildlife as a potential reservoir for animal and human anaplasmosis. However, further work is needed in order to evaluate the role of Korean water deer as a host/reservoir host of *A. capra*.

## Background

The cosmopolitan genus *Anaplasma* includes six species of Gram-negative obligate intracellular bacteria that are transmitted by ticks to a wide range of animals, including humans [1–5], resulting in considerable economic losses in the livestock industry and serious public health concerns [6, 7]. *Anaplasma phagocytophilum*, *A*. *ovis* and recently reported *A. capra* are human pathogens [8–12], whereas other species in the genus have no known zoonotic potential. However, *A. platys* may have zoonotic potential after frequent reports of human infection [13, 14].

The provisional name *Anaplasma capra* was assigned after its initial characterization in goats (*Capra aegagrus hircus*) in China [12]. Later, it was isolated from sheep, goats and cattle in different geographical regions [15–19] as well as from various tick species (*Haemaphysalis qinghaiensis*, *H. longicornis*, *Ixodes persulcatus*) [12, 20–23]. Infection of *A. capra* was also reported in six wild animals in China including three takins (*Budorcas taxicolor*), two Reeves’s muntjacs (*Muntiacus reevesi*) and one forest musk deer (*Moschus berezovskii*) [24].

*Anaplasma* species usually parasitize bone marrow-derived elements, including neutrophils (*A. phagocytophilum*), erythrocytes (*A. marginale*, *A. centrale* and *A. ovis*), monocytes (*A. bovis*) and platelets (*A. platys*) [7, 9, 10, 12]. However, *A. capra* seems to infect endothelial cells, rendering its microscopic detection in blood smears unreliable [12, 15]. In humans, the disease caused by *A. capra* is generally characterized by undifferentiated fever, headache, malaise, dizziness, myalgia and chills, with potential progression to CNS involvement and cerebrospinal fluid pleocytosis [12].

Although different *Anaplasma* species have been detected in wildlife [23–29], little is known about the prevalence and genetic identity of *A. capra* in these animals in Korea. Using molecular tools, the present study aimed at investigating the occurrence and characterizing the genetic profile of this pathogen in wildlife in the Republic of Korea.

## Methods

### Collection of samples

Chungbuk Wildlife Center is located in Cheongju city, Chungcheongbuk-do province in the Republic of Korea (36°38′13.99ʺN, 127°29′22.99ʺE). The center receives terrestrial and avian wild animals for purposes of treatment from sickness/injuries and/or rehabilitation. Blood samples are collected for diagnosis and treatment of wildlife referred to the Chungbuk Wildlife Center. Blood samples are archived in EDTA-treated tubes and stored at – 80 °C. A total of 253 blood samples including 198 from Korean water deer (*Hydropotes inermis argyropus*), 53 from raccoon dogs (*Nyctereutes procyonoides*) and one sample each from a leopard cat (*Prionailurus bengalensis*) and a roe deer (*Capreolus pygargus*), collected from January 2015 to June 2018, were used.

### DNA extraction and PCR amplification

Frozen blood samples were thawed at room temperature and genomic DNA was extracted from 200 µl of blood using a Magpurix® Blood Kit and Magpurix® 12s automated nucleic acid purification system (Zinexts Life Science Corp., Taipei, Taiwan), according to the manufacturer’s recommendations. DNA preparations were tested for the presence of *Anaplasma* species by PCR/sequence analysis of 429 bp of the *16S* rRNA gene as described previously [30]. *Anaplasma capra*-positive isolates were genetically profiled by the amplification of a longer fragment of *16S* rRNA (*rrs*) gene as well as partial sequences of citrate synthase (*gltA*), heat-shock protein (*groEL*), major surface protein 2 (*msp*2) and major surface protein 4 (*msp*4) genes as described previously (Table 1). Amplified fragments were electrophoresed on 1.2% gel loaded with EcoDye™ stain (BIOFACT, Daejeon, Korea) and visualized using UV light.

**Table 1:** PCR primers and conditions used in this study

Target gene	Primer name	Primer sequence (5′-3′)	Annealing T (°C)	Target size (bp)	Reference
*rrs*	Forward	TTGAGAGTTTGATCCTGGCTCAGAACG	57	1499	[12]
	Reverse	WAAGGWGGTAATCCAGC			
*gltA*	Outer F	GCGATTTTAGAGTGYGGAGATTG	55	1031	[12]
	Outer R	TACAATACCGGAGTAAAAGTCAA			
	Inner F	TCATCTCCTGTTGCACGGTGCCC	60	594	[21]
	Inner R	CTCTGAATGAACATGCCCACCCT			
*groEL*	Forward	GCGAGGCGTTAGACAAGTCCATT	58	1129	[12]
	Reverse	TCCAGAGATGCAAGCGTGTATAG			
*msp*2	Outer F	GCGTGTTGATGGCTCTGGT	52	1089	[12]
	Outer R	ACCAGTATCCTTATTTTTACC			
	Inner F	GAGTGCACCAGAGCCTAGAA	56	801	This study
	Inner R	TCACCATCACCAAGCACTCT			
*msp*4	Outer F	CAGTCTGCGCCTGCTCCCTAC	55	757	[12]
	Outer R	AGGAATCTTGCTCCAAGGTTA			
	Inner F	GGGTTCTGATATGGCATCTTC	56	656	[15]
	Inner R	GGGAAATGTCCTTATAGGATTCG			

*Abbreviations*: *rrs*, *16S* rRNA; *gltA*, citrate synthase; *groEL*, heat-shock protein; *msp*2, major surface protein 2; *msp*4, major surface protein 4; T, temperature

### DNA sequence analysis

The PCR products (*rrs* and *groEL*) or secondary PCR product (for other gene markers) were purified and sequenced, either directly or after cloning in the pGEM-T vector (Promega, Madison, WI, USA), in both directions. Generated sequences were assembled using ChromasPro v.2.1.8 (https://technelysium.com.au/wp/chromaspro/).

### Phylogenetic analysis

The obtained sequences from each genetic locus were aligned with each other and reference sequences, available in GenBank (https://www.ncbi.nlm.nih.gov/), using ClustalX (http://www.clustal.org/) to determine the identity of *Anaplasma* spp. Evolutionary relationships were inferred based on partial sequences of *16S* rRNA, citrate synthase (*gltA*), heat-shock protein (*groEL*), major surface protein 2 (*msp*2) and major surface protein 4 (*msp*4) genes using the maximum likelihood (ML) method implemented in MEGA7 (http://www.megasoftware.net/). The ML phylogenetic analysis was conducted using the Kimura 2-parameter model and 1000 bootstrap replicates. The ML tree was rooted against the nucleotide sequences L36221 (*Rickettsia typhi*), KY124257 (*Rickettsia parkeri*), U96733 (*Rickettsia rickettsii*) and BDDN01000175 (*Ehrlichia ruminantium*) for *16S* rRNA, *gltA*, *groEL* and *msp*4 gene markers, respectively.

## Results

The overall infection rate of *A. capra* in tested animals was 13.8% (35/253); however, samples from raccoon dogs (*n* = 53), leopard cat (*n* = 1) and roe deer (*n* = 1) were negative. The infection rate in KWD was 17.7% (35/198), distributed as follows: 24.6% (14/57) in 2015; 13.2% (5/38) in 2016; 17.3% (14/81) in 2017; and 9.1% (2/22) in 2018 (Table 2).

**Table 2:** Distribution of samples and prevalence of *A. capra* in animal species

Year	Korean water deer		Raccoon dog		Other animals	Number infected	Total number	Infection rate (%)
	Infected	Not infected	Infected	Not infected	Not infected			
2015	14	43	0	21	0	14	78	18.0
2016	5	33	0	5	0	5	43	11.6
2017	14	67	0	18	LC (1)	14	100	14.0
2018	2	20	0	9	RD (1)	2	32	6.3
Total number	35	163	0	53	2	35	253	13.8^a^
Infection rate/species (%)	17.7		0		0			

^a^Overall infection rate

*Abbreviations*: LC, leopard cat (*Prionailurus bengalensis*); RD, roe deer (*Capreolus pygargus*)

**Fig. 1:**
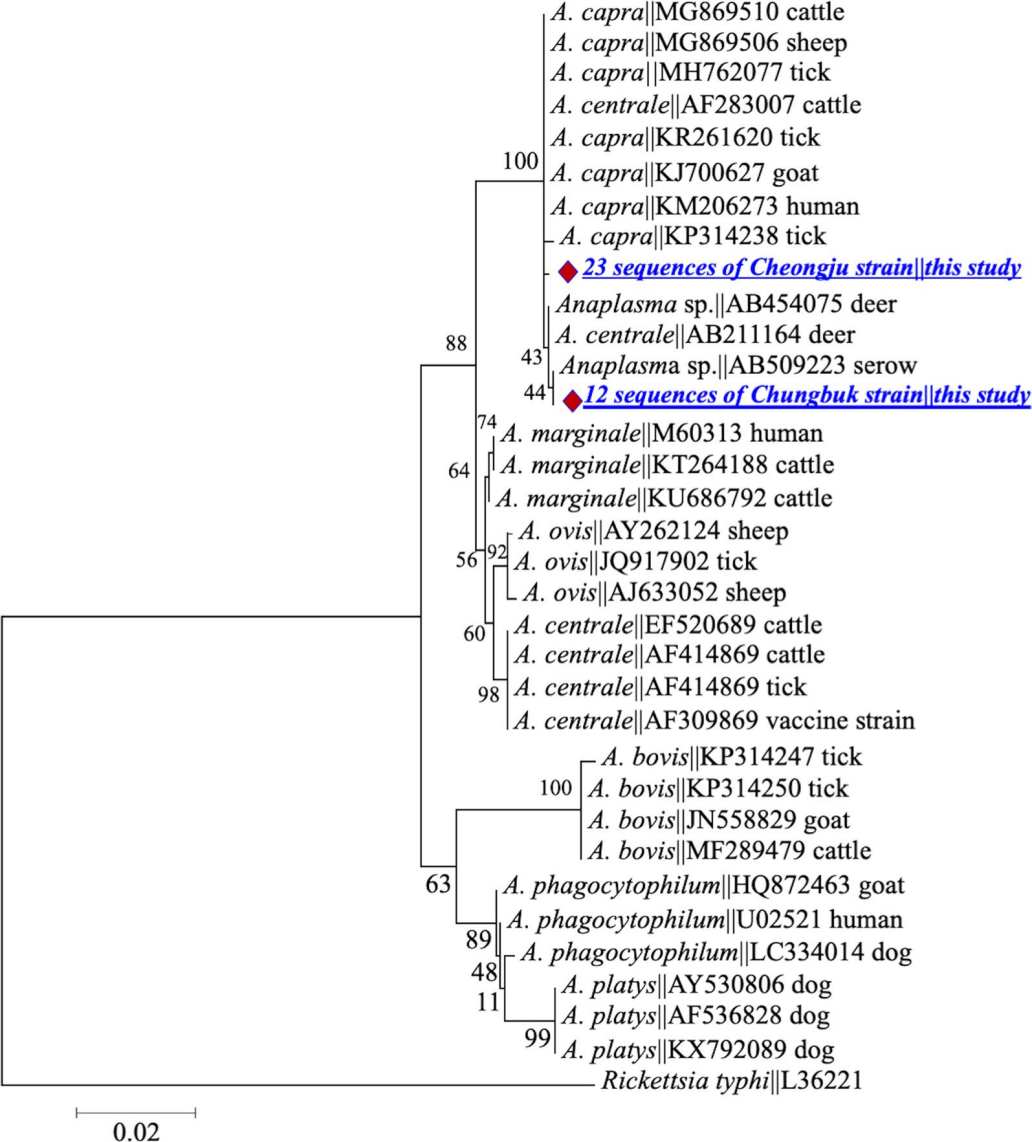
Maximum-likelihood phylogenetic trees of *Anaplasma* species based on partial sequences of *16S* rRNA gene. The tree was constructed using MEGA7 with the Kimura 2-parameter model. The newly generated sequences are indicated by diamonds. The numbers at nodes represent bootstrap values. The scale-bar represents the number of nucleotide substitutions per site

Molecular and phylogenetic analyses indicated to occurrence of two genetically distinct strains [named Cheongju (23 isolates) and Chungbuk (12 isolates)] of this pathogen. Sequences obtained from both strains were similar to those derived from *A. capra* from goats, sheep, cattle, ticks and humans; however, they had striking genetic differences, suggesting that they are novel strains. Sequences of the *rrs* gene fragment of both strains showed an identity of ~ 99.5% with counterparts in database and clustered in the clade of *A. capra* from different hosts (Fig. 1). Both strains had single nucleotide polymorphisms (SNPs), resulting in four genotypes at this gene locus. Phylogenetic analysis revealed that three sequences designated *A. centrale* (GenBank: AB211164, AF283007 and GU064903) and two sequences designated *Anaplasma* spp. (GenBank: AB454075 and AB509223) clustered within the *A. capra* clade, even though other *A. centrale* sequences from different hosts and geographical regions formed separate clusters in the ML tree.

**Fig. 2:**
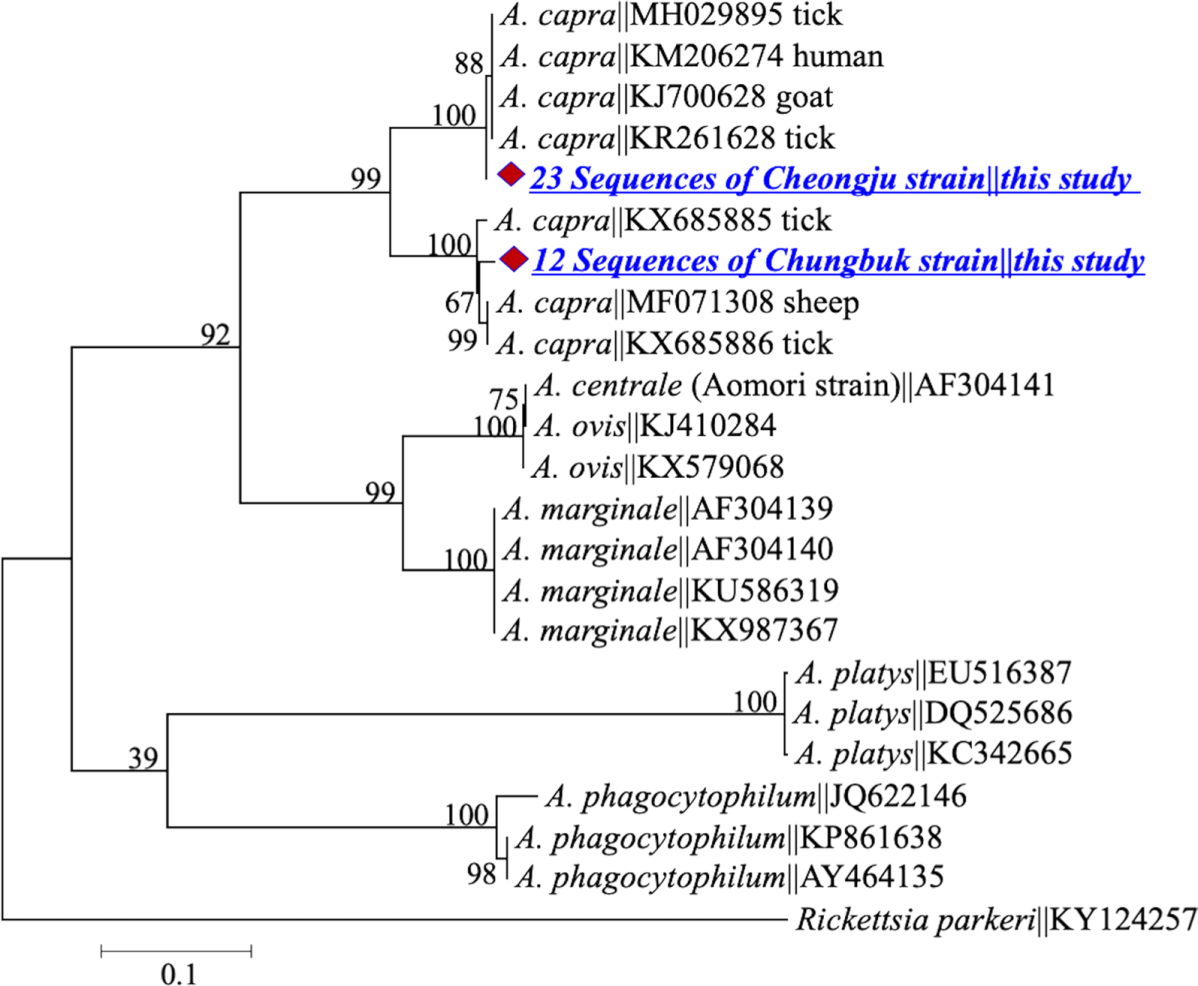
Maximum-likelihood phylogenetic trees of *Anaplasma* species based on partial sequences of *gltA* gene. The tree was constructed using MEGA7 with the Kimura 2-parameter model. The newly generated sequences are indicated by diamonds. The numbers at nodes represent bootstrap values. The scale-bar represents the number of nucleotide substitutions per site

**Fig. 3:**
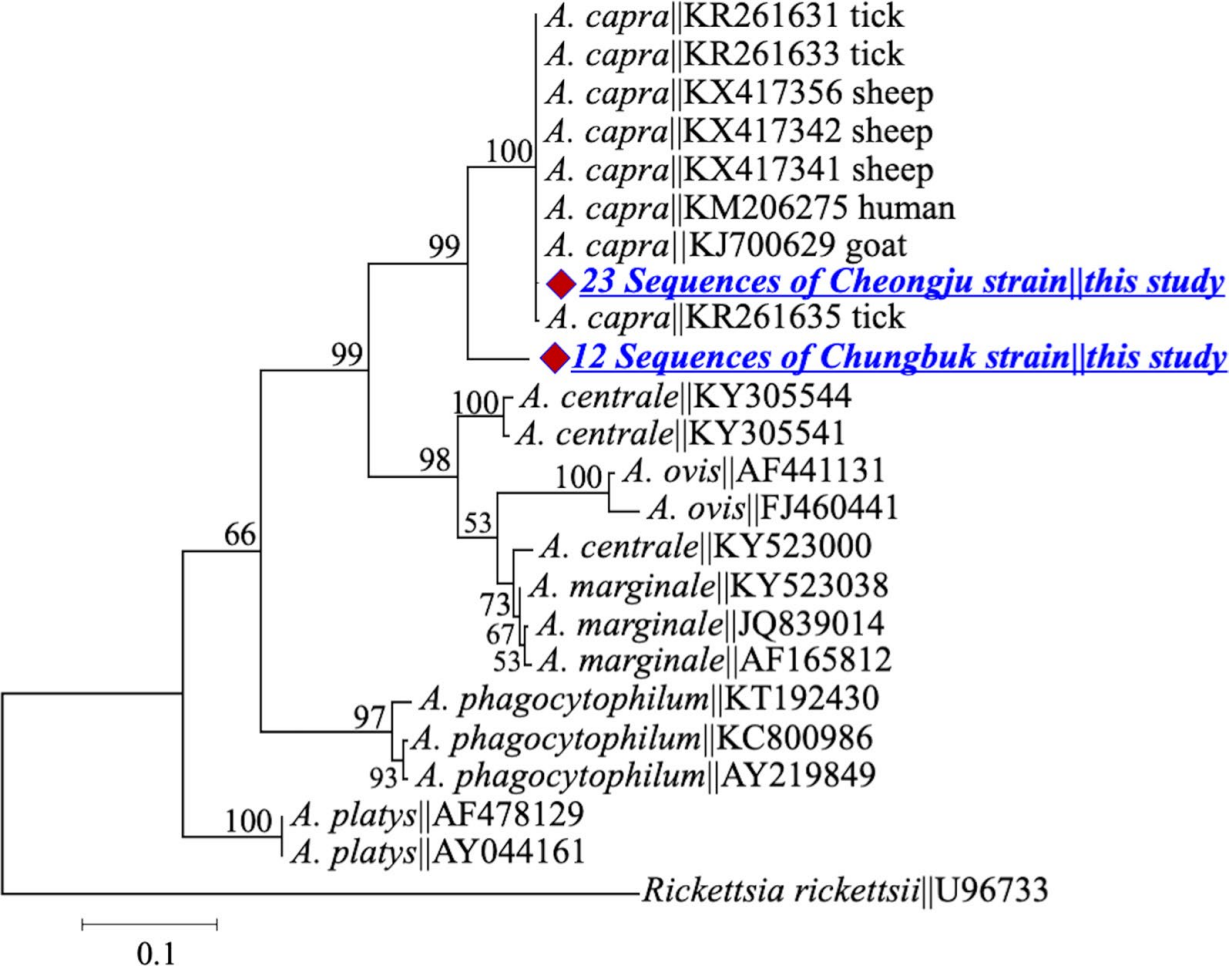
Maximum-likelihood phylogenetic trees of *Anaplasma* species based on partial sequences of *groEL* gene. The tree was constructed using MEGA7 with the Kimura 2-parameter model. The newly generated sequences are indicated by diamonds. The numbers at nodes represent bootstrap values. The scale-bar represents the number of nucleotide substitutions per site

**Fig. 4:**
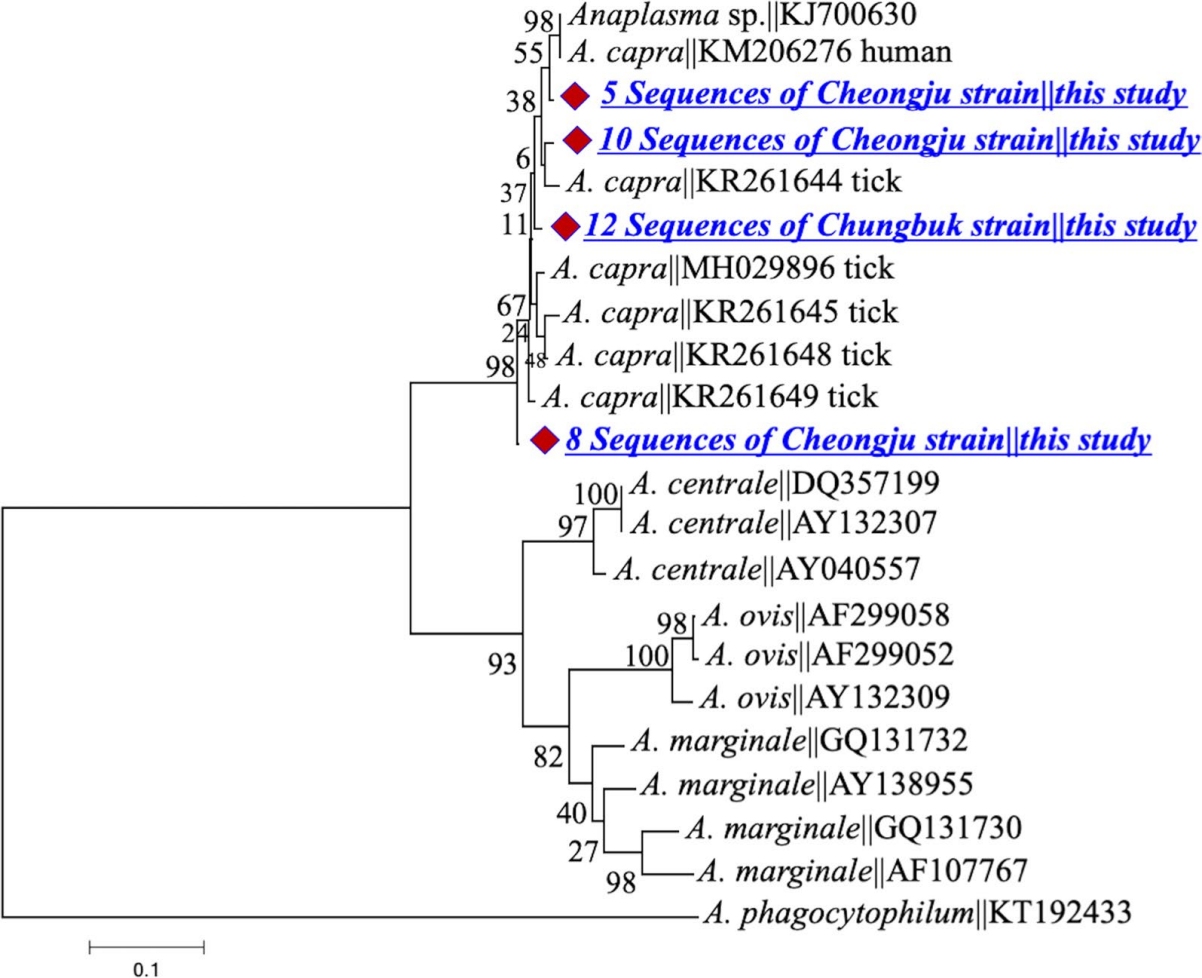
Maximum-likelihood phylogenetic trees of *Anaplasma* species based on partial sequences of *msp2* gene. The tree was constructed using MEGA7 with the Kimura 2-parameter model. The newly generated sequences are indicated by diamonds. The numbers at nodes represent bootstrap values. The scale-bar represents the number of nucleotide substitutions per site

**Fig. 5:**
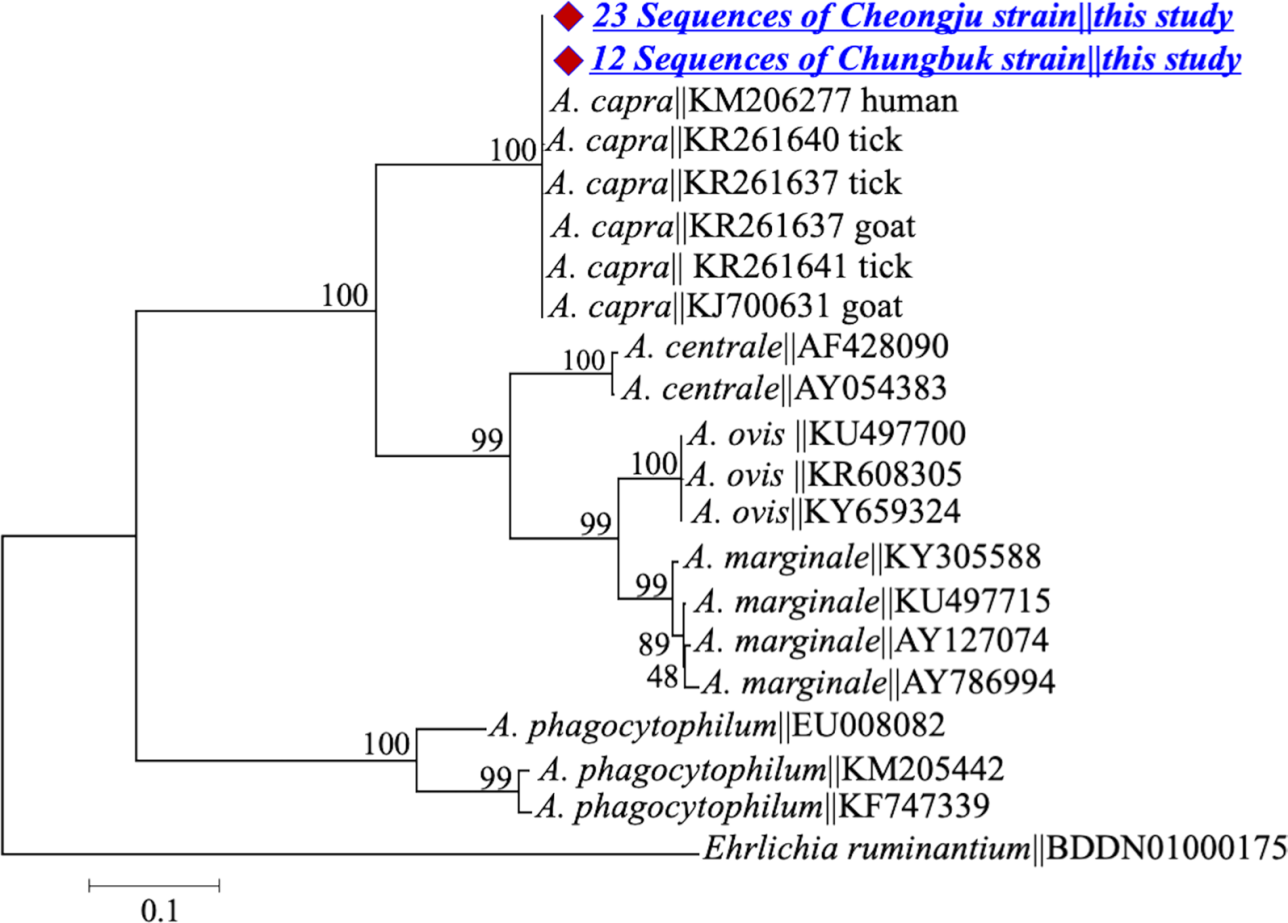
Maximum-likelihood phylogenetic trees of *Anaplasma* species based on partial sequences of *msp4* gene. The tree was constructed using MEGA7 with the Kimura 2-parameter model. The newly generated sequences are indicated by diamonds. The numbers at nodes represent bootstrap values. The scale-bar represents the number of nucleotide substitutions per site

The *gltA* gene of the Cheongju strain shared a similarity of 99.5% (with two substitutions, A/G at position 456 and T/C at position 533) with *gltA* sequences KM206274, KJ700628 and MH029895 isolated from a human, goat and tick, respectively [12, 23]. Sequences of the Chungbuk strain showed a similarity of 98–99% with KX685885, KX685886 and MF071308 of *A. capra* from ticks and sheep [13, 19]. Both strains clustered with their homologous sequences in the *A. capra* clade (Fig. 2). *groEL* gene sequences derived from the Cheongju strain shared a similarity of 99% (one substitution) with their counterparts from humans (GenBank: KM206275), goats (GenBank: KJ700629), sheep (GenBank: KX417356) and ticks (GenBank: KR261633 and KR261635), whereas sequences from the Chungbuk strain shared a similarity of 91% with the reference sequences (Fig. 3). The *msp*2 sequences showed extensive intra- and inter-sequence variations, including multiple InDels and single nucleotide substitutions; however, all sequences remained clustered in the *A. capra* clade (Fig. 4). A hypervariable stretch was detected between positions 285 and 414 of the generated sequences (corresponding to positions 550 and 679 in the reference sequence KM206276 of *A. capra* from humans). The *msp*4 sequences were identical in the two strains and showed an identity of 100% with those from humans (GenBank: KM206277) and ticks (GenBank: KR261637 and KR261640) (Fig. 5).

## Discussion

Wild animals act as reservoirs for a wide range of pathogens [31–33]. The emergence of infectious disease agents of wildlife origin is a prominent challenge to public health and the livestock industry [34–36]. *Anaplasma capra* has recently been isolated from human patients in China with non-specific clinical manifestations, with potential progression to CNS complications, suggesting that this species could pose a substantial threat to public health [12, 37]. We detected *A. capra* DNA in blood samples of 35 out of 198 KWD (17.7% infection rate) at the Chungbuk Wildlife Center, Korea. Epidemiological data for this pathogen in wildlife are lacking in Korea; however, our findings are similar to those obtained from wildlife (five takins, three Himalayan gorals, three Reeves’s muntjacs, one forest musk deer and one wild boar) in China [24]. In addition, a low percentage of infection rate was reported cattle, sheep and goats in China, Sweden and Korea [15–19], indicating that *A. capra* has a broad host range. Occurrence of infection during the study period from 2015 to 2018 indicates the persistence of the infection in KWD, suggesting that the species may act as a reservoir for this pathogen. However, it is difficult to explain the negative results from raccoon dogs in the present study. This may be attributed to persistent infection making the pathogen below detectable level in the blood of these animals. In support of this view, *A. capra* has been reported to infect endothelial cells [12, 15], making its detection in the blood possible in case of considerable bacteremia or released endothelial cells, resembling *Rickettsia* species [12]. Furthermore, the sample size and species and/or the age of animals may play a role in these findings. Further investigations are needed to clarify these points.

Our genetic profiling results indicated that the newly generated *16S* rRNA gene sequences shared a homology of > 99.5% with sequences of *A. capra* strains from humans, sheep, goats, cattle and ticks [12, 15, 16, 18, 20, 22], suggesting that they likely are within the same species of bacteria [38, 39]. Clustering of sequences named *A. centrale* from deer (*Cervus nippon nippon*) (GenBank: AB211164) and cattle (GenBank: AF283007) in Japan and from ticks (*Haemaphysalis longicornis*) in Korea (GenBank: GU064903), as well as *Anaplasma* spp. from deer (GenBank: AB454075; direct submission) and Japanese serow (*Capricornis crispus*) (GenBank: AB509223) in the same clade of *A. capra* indicate a close phylogenetic relationship. The clustering pattern of these sequences in the *A. capra* clade does not support their assignment as sister taxa and suggests that these isolates are in fact *A. capra* [15, 25] and may need re-description, since these sequences were more related to Chungbuk strain in the *A. capra* clade.

The results obtained using different gene markers showed considerable sequence variation, suggesting that *A. capra* has a high degree of genetic diversity. Notably, extensive genetic variation was detected in *msp*2. Consistent with our results, sequence variation at the studied gene markers has been reported previously among isolates from ticks, sheep and goats [12, 15–18, 20–25]. Similarly, genetic variation is common in other *Anaplasma* species [40–45]. Although genetic diversity is reportedly related to infectivity, virulence, pathogenicity, niche preference, immune evasion, and/or host adaptability [46–49], this has not been established in *A. capra* and further studies are needed to evaluate these relationships.

Due to the extinction of natural predators, the KWD is thriving in Korea and has been designated as “harmful wildlife” by the Ministry of Environment in 1994 owing to harmful interactions with humans and their properties. This close interaction poses substantial threats to domestic animals and human health in Korea. This study was limited by analyzing samples from one geographical area and few animal species, which my lead to biases in the results. A large-scale study is underway to fully elucidate the host range of wildlife, vector ticks, pathogenicity and geographical distribution of this organism in Korea.

## Conclusions

To our knowledge, the results presented herein provide the first evidence for the presence of *A*. *capra* in Korean water deer in Korea. As an emerging human pathogen, the detection of *A. capra* in deer provides insight into the role of wildlife as a potential reservoir for human anaplasmosis. Furthermore, the obtained results expand the known geographical and host range of the *Anaplasma capra*.

## Data Availability

Data supporting the conclusions of this article are provided within the article. The nucleotide sequences generated in this study are available in the GenBank database under the accession numbers LC432092–LC432126 (*rrs*), LC432127–LC432161 (*gltA*), LC432162–LC432196 (*groEL*), LC432232–LC432266 (*msp*2) and LC432197–LC432231 (*msp*4).

## Abbreviations

*rrs*: *16S* rRNA
*gltA*: citrate synthase
*groEL*: heat-shock protein
*msp*2: major surface protein 2
*msp*4: major surface protein 4 gene
KWD: Korean water deer (*Hydropotes inermis argyropus*)
ML: maximum likelihood
